# AKT and PERP Show Higher Expression in Precancerous than in Malignant Skin Neoplasms: Profiling in an Animal Model of Sequential Skin Carcinogenesis

**DOI:** 10.3390/jpm14080790

**Published:** 2024-07-25

**Authors:** Efstathia Vairaktari, Alexander Schramm, Georgia Vairaktari, Spyridoula Derka, Frank Wilde, Andreas Sakkas, Christos Yapijakis, Maria Kouri, Athanasios Balakas, Andreas Lazaris, Marcel Ebeling, Stavros Vassiliou

**Affiliations:** 1 Department of Oral and Maxillofacial Surgery, University General Hospital Attikon, School of Medicine, National and Kapodistrian University of Athens, 11527 Athens, Greece; 2 Department of Oral and Maxillofacial Surgery, University Hospital Ulm, Albert-Einstein-Allee 10, 89081 Ulm, Germany; 3 Department of Oral and Plastic Maxillofacial Surgery, Military Hospital Ulm, Academic Hospital of the University of Ulm, Oberer Eselsberg 40, 89081 Ulm, Germany; 4 Unit of Orofacial Genetics, University Research Institute for the Study of Genetic and Malignant Disorders in Childhood, School of Medicine, National and Kapodistrian University of Athens, 11527 Athens, Greece; 5 Department of Oral Medicine & Pathology and Hospital Dentistry, School of Dentistry, National and Kapodistrian University of Athens, 11527 Athens, Greece; 6 Department of Pathology, School of Medicine, National and Kapodistrian University of Athens, 11527 Athens, Greece

**Keywords:** skin cancer, experimental carcinogenesis, PERP, Akt, immunohistochemistry

## Abstract

The primary aim of this study was to evaluate the activation of the PERP and Akt oncogenes in the induction of skin cancer in FVB/N mice by a stepwise chemical process. Forty four-week-old female FVB/N mice were randomly divided into a control group (n = 8) and two experimental groups (group A: n = 16, group B: n = 16). In the study, the groups were subjected to a two-stage carcinogenesis procedure. This consisted of an initial application of 97.4 nmol DMBA to shaved skin on the back, followed by applications of 32.4 nmol TPA after thirteen weeks for group A and after twenty weeks for group B. The control group received no treatment. Skin conditions were monitored weekly for tumor development. At the end of the experiment, the animals were euthanized for further tissue sampling. Examination of the skin lesions in the experimental groups showed a correlation with tumor progression, ranging from dysplasia to carcinoma. Tumor samples were examined both histologically and immunohistochemically. Notably, and PERP expression was higher in precancerous than in malignant tumors. The differences in expression between precancerous and benign tumors provide further evidence of a role for PERP and Akt in the transition from benign to malignant states. Our findings underscore the critical roles of PERP and Akt in the pathogenesis of skin cancer and suggest their potential as biomarkers for early detection and targets for therapeutic intervention.

## 1. Introduction

Malignant neoplasms of the skin represent one of the most significant challenges in public health [1,2,3]. The most frequently diagnosed malignant neoplasms have had a rising incidence worldwide for decades [4,5,6]. This can be explained primarily by increased exposure to ultraviolet light, genetic predispositions, and environmental influences [7,8]. The three main histological types of skin cancer are basal cell carcinoma (BCC), squamous cell carcinoma (SCC), and melanoma [3]. These can be distinguished on the basis of their typical, distinct etiology, histopathological features, and clinical behavior [9,10].

Non-melanoma skin cancer (NMSC), which includes BCC and SCC, accounts for most skin cancer cases in percentage terms [4]. BCC is the neoplasm with the highest prevalence, typically characterized by its slow growth and very low metastatic rate. Although SCC is less common than BCC, it has a significantly higher risk of metastasis and a more aggressive growth pattern. In comparison, melanomas have a high metastatic potential and mortality rate [3]. All three histological types share commonalities in their underlying pathogenesis, including interactions among genetic mutations, environmental influences, and misdirected immune responses [11].

Recent research into molecular analyses has identified a number of pathways that are involved in the development of skin cancer. In particular, PERP (p53 apoptosis effector related to PMP-22) and Akt (protein kinase B) have become the focus of research due to their involvement in apoptosis, proliferation, and cell survival [12,13].

PERP is a tetraspan membrane protein encoded by the PERP gene, which is a transcriptional target of the tumor suppressor p53. It is primarily known for its role in mediating p53-dependent apoptosis [13]. PERP is localized to desmosomes, and alterations in PERP function have been shown in various cancer types [14,15,16,17]. In normal physiological conditions, PERP ensures the integrity of epithelial tissue and the adhesion of cells to cells. In the event of DNA damage, p53 is activated, which induces PERP expression, leading ultimately to apoptosis of potentially malignant cells. Dysregulation of PERP, for example through genetic mutations, epigenetic changes, or changes in p53 signaling, can reduce apoptosis and promote tumor development [18,19,20]. The loss of PERP function not only leads to an impairment of apoptotic capacity but also to a disruption of cell adhesion [21]. This promotes the ability of the cells to detach and their metastatic potential [11].

Akt, also known as protein kinase B, is a serine/threonine kinase that plays a vital role in a range of cellular processes, including metabolism, proliferation, survival, and growth [22,23,24]. It is a central component of the phosphatidylinositol 3-kinase (PI3K)/Akt signaling pathway, which is activated by a plethora of growth factors and extracellular signals. During activation, Akt phosphorylates, another substrate that regulates cell survival, cell cycle progression, and metabolism [25,26,27]. The phosphatidylinositol 3-kinase (PI3K)/Akt signaling pathway is frequently dysregulated in cancer, resulting in enhanced cell survival, proliferation, and resistance to apoptosis. Mutations in PI3K, the loss of the tumor suppressor PTEN (phosphatase and tensin homologue), and amplifications or mutations in Akt itself can lead to persistent activation of the signaling pathway [28,29]. Impaired Akt signaling is associated with the development of both melanoma and non-melanoma skin cancers [29]. Increased Akt activity is associated with increased cell proliferation, inhibition of apoptosis, angiogenesis, and the spread of metastases [12,30,31].

The interaction of PERP and Akt represents a significant scientific field, given that the two genes play opposing roles in apoptosis and survival. While PERP acts as a pro-apoptotic and tumor-suppressive molecule, Akt acts as a pro-survival and oncogenic kinase. It is important to maintain a balance between these signaling pathways to ensure a stable cellular homeostasis and prevent malignant transformation.

In the context of skin cancer, alterations in PERP and Akt expression and function have profound effects on disease progression. A reduction in PERP expression, which is frequently observed in various skin neoplasms, impairs apoptotic responses mediated by p53 and allows damaged cells to evade cell death. Hyperactivation of Akt signaling can further inhibit apoptotic pathways and promote cell survival and proliferation, creating a favorable environment for tumor growth and progression. Restoration of PERP function or enhancement of its expression could restore apoptotic mechanisms and suppress tumor growth. Consequently, inhibition of Akt activity by specific inhibitors or modulation of upstream regulators may lead to a reduction in survival signaling and sensitization of cancer cells to apoptosis.

The primary aim of this study was to investigate the differential expression patterns of PERP and Akt genes in various histopathological types of skin neoplasms in sequential chemical skin carcinogenesis. We hypothesize that alterations in PERP and Akt expression contribute to the distinct biological behaviors. To achieve this objective, we conducted a comprehensive immunohistochemical analysis of PERP and Akt expression in a series of chemically induced skin neoplasms, including benign, precancerous, and malignant lesions. By correlating gene expression profiles with histopathological features, we aim to provide insights into the molecular underpinnings of skin carcinogenesis and identify potential avenues for targeted interventions.

## 2. Materials and Methods

### 2.1. Institutional Review Board Statement

This study was conducted in accordance with the ethical standards of the institutional research committee and the 1964 Helsinki Declaration and its later amendments or comparable ethical standards. Ethical approval was obtained from the Bioethics committee of the School of Medicine at the National and Kapodistrian University of Athens (Approval Number: 6726/21-12-2015, Approval Date: 23 December 2015). The reporting was conducted in accordance with the recommendations of the Strengthening the Reporting of Observational Studies in Epidemiology (STROBE) initiative.

### 2.2. Animals

A total of 40 female FVB/N mice, aged four weeks and weighing approximately 100 g each, were obtained from the Hellenic Pasteur Institute in Athens, Greece. The FVB strain, which is known to be susceptible to the Friend leukemia virus B, was selected for its suitability in transgenic research and documented susceptibility to skin tumors. To avoid issues with male aggression, age-matched females were selected. Following a two-week period of acclimatization, the mice were randomly assigned to three groups: a control group (n = 8) and two experimental groups (n = 16 each). Rigorous randomization and adherence to ARRIVE guidelines were ensured. The mice were housed in groups of four, with each animal bearing an ear tattoo for identification purposes. They were maintained under a 12-h light/dark cycle, with access to appropriate bedding and food and water ad libitum. This approach ensured the welfare of the animals and the integrity of the scientific process.

### 2.3. Two-Stage Carcinogenesis Protocol

A total of 40 female FVB/N mice, aged four weeks, were included in the study after being found to be in the telogen phase. Groups A and B (n = 16 each) were subjected to a topical treatment with 7,12-dimethylbenz[a]anthracene (DMBA) followed by 12-O-tetradecanoyl phorbol-13-acetate (TPA) for 13 weeks (group A) and 20 weeks (group B). Group C (n = 8) served as the control group. A series of weekly examinations were conducted to observe the lesions. Dermatological assessments were conducted in accordance with the methodology proposed by Quintanilla et al. The animals were euthanized by an overdose of isoflurane. At fourteen weeks (Group A) or twenty-one weeks (Group B), excision of lesions was performed. The tumor sizes were approximately 1 cm. The tissue samples were evaluated in an impartial manner.

### 2.4. Histopathological Analysis

A total of 459 biopsy samples underwent fixation and embedding. The histological analysis involved staining with hematoxylin and eosin, as well as immunohistochemical detection of PERP and Akt gene products. The tissue profiles were classified into distinct categories, including normal, hyperkeratosis, hyperplasia, dysplasia (low grade, high grade), papilloma, in situ carcinoma, well-differentiated squamous cell carcinoma, and poorly differentiated squamous cell carcinoma. Each sample was subjected to a meticulous evaluation to ensure accurate categorization, thereby providing a detailed understanding of the tissue alterations.

### 2.5. Immunohistochemical Analysis

Tissue sections were incubated with antibodies against PERP and Akt, sourced from Thermo Fisher Scientific, in accordance with standard immunohistochemical methodology. The positive controls included mouse skin tissue with robust PERP expression and osteosarcoma tissue with strong Akt expression. The negative controls underwent parallel processing with phosphate-buffered saline (PBS) instead of the primary antibody. Two independent investigators, who were unaware of the samples’ identities, reviewed all the samples, thereby ensuring unbiased evaluation and reinforcing the study’s reliability and validity.

### 2.6. Statistical Analysis

Nominal variables are presented with absolute and relative frequencies (%), whereas continuous with mean, standard deviation, median, and interquartile range (IQR). The non-parametric Mann-Whitney test was used to compare the percentage of PERP and Akt positive expression in groups A and B with the control group as well as between the two groups, since the assumption of normality was not satisfied according to the Kolmogorov-Smirnov criterion and histograms. The Fisher’s Exact test was used to compare the percentage of PERP and Akt positive expression of normal histology with precancerous, benign, and malignant tumors. The differences among the percentages of PERP and Akt positive expression were evaluated via the McNemar test. All reported *p* values are two-tailed. Statistical significance was set to 5%, and analyses were conducted using STATA SE v18.

## 3. Results

A total of 459 biopsies were analyzed, comprising eight from the control group, 211 from Group A, and 240 from Group B. The histological status of each group is detailed in Table 1.

**Figure 1:**
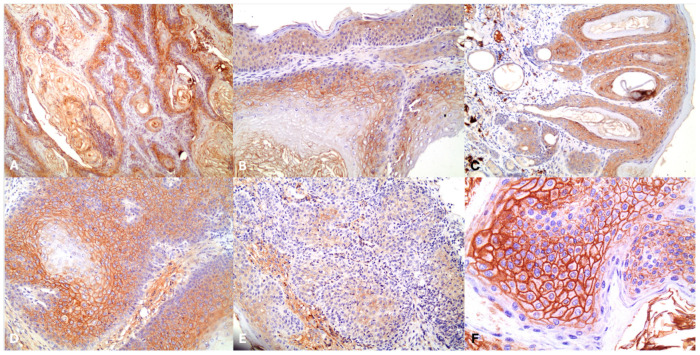
(**A**) PERP immunostaining in well-differentiated squamous cell carcinoma; (**B**) Selective PERP immunostaining; (**C**) PERP in hyperplastic epithelium; (**D**) Intense PERP immunostaining in hyperplastic squamous cells; (**E**) Decreased PERP immunostaining in severely dysplastic cells; (**F**) Increased PERP immunostaining in parabasal hyperplastic cells.

**Figure 2:**
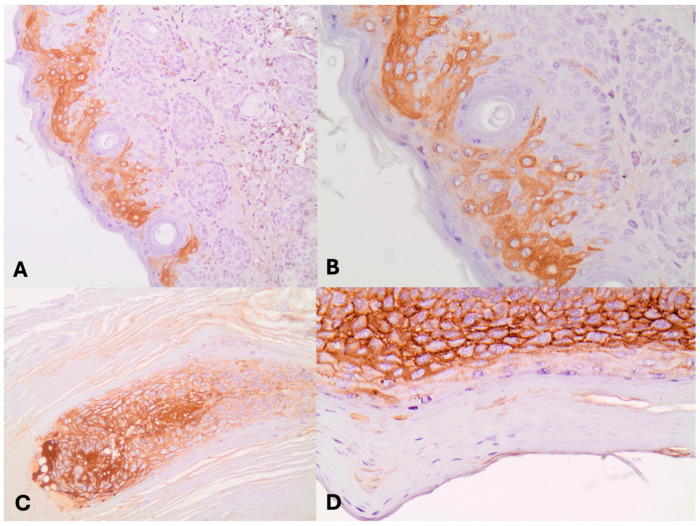
(**A**) Increased Akt cytoplasmic immunoreactivity in squamous cells (×200); (**B**) Increased Akt cytoplasmic immunoreactivity in squamous cells (×400); (**C**) Increased Akt immunopositivity in hyperplastic squamous cells (×200); (**D**) Increased Akt immunopositivity in hyperplastic squamous cells (×400).

Table 2 presents the percentage of PERP and Akt positive expression per animal across the groups. The control group exhibited a mean positive expression of 12.5% (standard deviation [SD] = 35.3%), while Group A demonstrated 56.4% (SD = 12.8%), and Group B exhibited 60.7% (SD = 17.2%) (Figure 1 and Figure 2).

The median percentage of positive PERP expression in Groups A and B was found to be significantly higher than that observed in the control group (*p* = 0.003 and *p* = 0.003, respectively), with no significant difference observed between Groups A and B (*p* = 0.431).

Regarding Akt, the mean positive expression was 0% (SD = 0%) in the control group, 28.1% (SD = 12.4%) in Group A, and 23.4% (SD = 11.2%) in Group B. Both Groups A and B exhibited significantly higher mean Akt positive expression compared to the control group (*p* < 0.001 for both), with no significant difference between Groups A and B (*p* = 0.584).

Table 3 presents the percentages of PERP and Akt positive expression according to histological status. The positive expression of PERP and Akt was found to be significantly higher in precancerous tumors than in normal histology (72.8% vs. 18.8%, *p* = 0.001 for PERP; 37.7% vs. 0.0%, *p* = 0.001 for Akt). Furthermore, significant differences were observed between PERP and Akt positive expression in precancerous (72.8% vs. 37.7%) and benign tumors (26.6% vs. 0.9%) (*p* = 0.001 for both comparisons). In summary, the proportion of tumors exhibiting PERP expression was significantly higher than that of Akt (57.3% vs. 26.4%, *p* < 0.001).

## 4. Discussion

This study provides a comprehensive analysis of PERP and Akt expression in chemically induced skin carcinogenesis, highlighting significant differences in gene expression across various histopathological stages of skin neoplasms. Our findings underscore the critical roles of PERP and Akt in the pathogenesis of skin cancer and suggest their potential as biomarkers for early detection and targets for therapeutic intervention.

The elevated expression of PERP and Akt in precancerous lesions compared to normal tissues especially highlights their involvement in early neoplastic changes. Additionally, the significant expression differences between precancerous and benign tumors indicate a potential role for PERP and Akt in the progression from benign to malignant states.

The elevated expression of PERP in groups A and B in comparison to the control group is noteworthy. The mean expression of PERP was found to be significantly higher in group A (56.4%) and group B (60.7%) than in the control group (12.5%) (*p* = 0.003). This indicates that PERP plays a pivotal role, particularly during the initial stages of chemically induced carcinogenesis of the skin. PERP is known to be a transcriptional target of p53 and thus is involved in apoptosis and cell-to-cell adhesion [18,19,20,21]. The elevated expression of PERP in response to chemical induction is likely to reflect an attempt by the cells to counteract the carcinogenic process by promoting apoptosis and maintaining epithelial integrity. Nevertheless, the elevated expression levels of PERP in the chemically treated groups also suggest the potential for dysregulation in its apoptotic function.

The lack of a significant difference in PERP expression between group A and group B (*p* = 0.431) indicates that once PERP is upregulated due to chemical stimulation, there is a plateau in expression, which may be explained by saturation or feedback mechanisms.

It has been demonstrated that mice lacking PERP in the skin exhibit resistance to papilloma development, displaying fewer and smaller papillomas compared to wild-type mice. The proliferation levels, apoptotic indices, and differentiation patterns in the skin of treated PERP-deficient and wild-type mice remain comparable. It is, therefore, proposed that the diminished tumor development observed in the absence of PERP may be explained by impaired adhesion due to aberrant desmosome assembly. These studies indicate that, in certain contexts, PERP is required for efficient carcinogenesis and suggest a role for intact cell-cell adhesion in supporting tumor development in these settings [32]. The study by Beaudry et al. examines the impact of desmosome loss on carcinogenesis by analyzing conditional knockout mice lacking PERP, a gene regulated by p53/p63 that is essential for desmosomes. In a UVB-induced squamous cell carcinoma model, PERP deficiency has been observed to promote both tumor initiation and progression. This is linked to the inactivation of PERP’s roles in apoptosis and cell–cell adhesion. Tumors lacking PERP exhibit a reduction in desmosomal components, while adherens junctions remain intact. This indicates that desmosome loss is a crucial factor in tumorigenesis. Similarly, human squamous cell carcinomas exhibit a loss of PERP expression yet retain adherens junctions, suggesting a relevant stage in cancer development. Gene expression profiling indicates that PERP loss results in the activation of inflammation-related genes, which may contribute to tumorigenesis [33].

Akt, a serine/threonine kinase involved in the PI3K/Akt signaling pathway, exhibited significantly higher expression in Groups A and B compared to the control group. The mean positive expression of Akt in Groups A and B (28.1% and 23.4%, respectively) was found to be significantly elevated relative to the control group (0%), with *p*-values of less than 0.001 for both comparisons.

Akt is known to support cell survival, proliferation, and growth by inhibiting the apoptosis process, and it has been described as being upregulated in various cancers [28,29,30,31]. The elevated Akt expression observed in our study lends support to the hypothesis that Akt promotes tumor progression through increased cellular survival and proliferation, in opposition to the pro-apoptotic mode of action of PERP.

The histopathological analysis further highlights the significance of PERP and Akt in skin carcinogenesis. Both genes showed significantly higher expression in precancerous lesions compared to normal tissue (PERP: 72.8% vs. 18.8%, *p* = 0.001; Akt: 37.7% vs. 0%, *p* = 0.001). This data suggests that the upregulation of these genes is an early event in the carcinogenic process, potentially serving as early biomarkers and therapy targets. The significant differences in expression between precancerous and benign tumors (PERP: 72.8% vs. 26.6%, *p* = 0.001; Akt: 37.7% vs. 0.9%, *p* = 0.001) further highlight their involvement in the transition from benign to malignant neoplasms.

It is notable that the overall proportion of tumors expressing PERP was significantly higher than those expressing Akt (57.3% vs. 26.4%, *p* < 0.001). This suggests that although both genes are important in the development of skin cancer, PERP may play a more important role in the early stages of tumor development. The higher expression of PERP may be an initial cellular response to carcinogenic stress aimed at inducing apoptosis and maintaining tissue integrity. However, as the carcinogenic process progresses, Akt-mediated survival pathways become more prominent, favoring tumor growth and progression. In a similar manner, both apoptosis and cell proliferation were observed to culminate in the early stages of sequential oral squamous cell carcinogenesis in a rodent model developed by our group [34].

The divergent expression patterns observed for PERP and Akt at different histopathological stages suggest potential therapeutic implications. Targeting PERP to enhance its apoptotic function may represent a promising strategy for early intervention in precancerous lesions. Conversely, inhibition of Akt activity could attenuate the survival and proliferation benefits provided by this signaling pathway, thereby suppressing tumor growth and progression.

However, considering the findings presented here, it is important to acknowledge the limitations of this study. The precise way PERP and Akt interact with one another in the mouse model and potentially influence one another remains unresolved. Furthermore, it is not possible to make any statements regarding the potential influence of other molecular pathways on the results presented here. A further limitation of this study is the small size of the experimental cohort, which consisted of only 40 mice. Consequently, while minor fluctuations or tendencies may be discernible, they may not be precisely indicative. Furthermore, the decision to exclusively use female mice, although potentially justified by specific research considerations, may limit the generalizability of the results.

Nevertheless, it is essential to interpret these findings with caution, given the limitations previously outlined. The obtained data provide evidence supporting the hypotheses of the involvement of PERP and Akt in various stages of tumorigenesis, including the initial oncogenic phases and advanced malignancies. Furthermore, both genes exhibit higher expression in precancerous than in malignant neoplasms. PERP appears to play a more prominent role in skin carcinogenesis. This may stimulate further research. Should these results be corroborated in future applications for the diagnosis, treatment, or prognosis of skin cancer, they may become of greater significance.

## 5. Conclusions

The significantly elevated expression of PERP and Akt in precancerous lesions in comparison to normal tissues highlights their involvement in the early stages of neoplastic transformation. The differences in expression between precancerous and benign tumors provide further evidence of the role of PERP and Akt in the transition from benign to malignant states. Further studies are required to fully evaluate the role of PERP and Akt in the pathogenesis of skin cancer.

## Figures and Tables

**Table 1:** Histological status of biopsies in the control and the two experimental groups.

	Group					
	Control		A		B	
	Ν	%	Ν	%	Ν	%
Normal	8	100.0	6	2.8	2	0.8
Precancerous	0	0.0	162	76.8	151	62.9
Benign tumors	0	0.0	34	16.1	79	32.9
Malignant tumors	0	0.0	9	4.3	8	3.3

**Table 2:** Percentages (%) of PERP and Akt positive cells in the control group and the two experimental groups.

	PERP				Akt			
	Control Group	Group A	Group Β	*p*-Value + (Group A vs. Group B)	Control Group	Group A	Group Β	*p*-Value + (Group A vs. Group B)
Mice 1	0.0%	61.9%	36.4%		0.0%	42.9%	36.4%	
Mice 2	0.0%	53.8%	23.5%		0.0%	53.8%	23.5%	
Mice 3	0.0%	56.3%	60.0%		0.0%	25.0%	0.0%	
Mice 4	100.0%	73.3%	66.7%		0.0%	40.0%	11.1%	
Mice 5	0.0%	50.0%	72.0%		0.0%	22.7%	32.0%	
Mice 6	0.0%	50.0%	57.1%		0.0%	21.4%	25.7%	
Mice 7	0.0%	68.8%	33.3%		0.0%	31.3%	26.7%	
Mice 8	0.0%	62.5%	52.0%		0.0%	50.0%	20.0%	
Mice 9		37.5%	75.0%			12.5%	25.0%	
Mice 10		52.9%	66.7%			29.4%	8.3%	
Mice 11		88.9%	75.0%			22.2%	12.5%	
Mice 12		55.6%	58.3%			16.7%	16.7%	
Mice 13		40.0%	87.5%			20.0%	37.5%	
Mice 14		50.0%	68.8%			25.0%	37.5%	
Mice 15		57.1%	75.0%			14.3%	33.3%	
Mice 16		44.4%	64.3%			22.2%	28.6%	
Mean% (SD)	12.5 (35.3)	56.4 (12.8)	60.7 (17.2)		0.0 (0.0)	28.1 (12.4)	23.4 (11.2)	
Median% (IQR)	0.0 (0.0–0.0)	54.7 (50–62.2)	65.5 (54.6–73.5)	0.431	0.0 (0.0–0.0)	23.8 (20.7–35.6)	25.4 (14.6–32.7)	0.584
*p* + (comparison with control group)	-	0.003	0.003		-	<0.001	<0.001	

+ *p*-value from Mann-Whitney test.

**Table 3:** Percentages of PERP and Akt positive cells according to histological status.

	PERP Positive Cells		Akt Positive Cells		
	N (%)	*p*-Value +	N (%)	*p*-Value +	*p*-Value * (PERP vs. Akt)
Normal	3/16 (18.8%)		0/16 (0.0%)		0.083
Precancerous	228/313 (72.8%)	0.001	118/313 (37.7%)	0.001	0.001
Benign tumors	30/113 (26.6%)	0.503	1/113 (0.9%)	0.706	0.001
Malignant tumors	2/17 (11.8%)	0.656	2/17 (11.8%)	0.485	0.654
Total Sample	263/459 (57.3%)		121/459 (26.4%)		<0.001

+ *p*-value from Fisher’s Exact test for comparison with normal histological status; * *p*-value from the McNemar test for comparison between PERP and Akt % of positive cells.

## Data Availability

The data is available from the corresponding author on reasonable request.
